# Cascade Semantic Segmentation by a Convolutional Neural Network in Combination with Image Super-Euclidean Pixels Processing for SARS-CoV-2 Microscopy Images

**DOI:** 10.3390/v18060592

**Published:** 2026-05-24

**Authors:** Santiago Tello-Mijares, Francisco Flores, Fomuy Woo

**Affiliations:** 1 Instituto Tecnológico de la Laguna, Tecnológico Nacional de México Campus Laguna, Torreón 27150, Mexico; fgfloresg@lalaguna.tecnm.mx; 2 Medical Familiar Unit, Instituto de Seguridad y Servicios Sociales de Los Trabajadores del Estado, Torreón 27268, Mexico; luisa.fomuy@issste.gob.mx

**Keywords:** convolutional neural networks, deep learning, JET colour model, SARS-CoV-2, semantic segmentation, super-Euclidean pixels

## Abstract

Although SARS-CoV-2 has been extensively studied from clinical, virological, and diagnostic perspectives, the problem of accurate automatic semantic segmentation of SARS-CoV-2 particles in electron microscopy images remains inadequately explored. Existing studies have largely focused on virus detection, classification, morphometry, or conventional image analysis, while comparatively little attention has been paid to pixel-level delineation of viral structures using specialised deep learning segmentation frameworks. To address this gap, we propose here a deep learning system based on convolutional neural networks (CNNs) combined with image processing techniques to establish semantic segmentation tools for the automatic identification of SARS-CoV-2. Our approach utilises the super-Euclidean pixels method as an intermediate layer within the CNN for semantic segmentation. We then compare its performance against the gradient vector flow (GVF) and Poisson inverse gradient (PIG) segmenters. The proposed CNN model surpassed the traditional GVF and PIG segmentation models, achieving the following metrics (mean ± variance): Dice similarity coefficient (DSC) = 0.9345 ± 0.0006; intersection over union (IoU) = 0.8782 ± 0.0018; sensitivity/true positive rate (TPR) = 0.9373 ± 0.0018; specificity/true negative rate (SPC) = 0.9517 ± 0.0012; accuracy = 0.9449 ± 0.0004; area under the ROC curve (AUC) = 0.9446 ± 0.0431; and Cohen’s Kappa = 0.9137 ± 0.0011. This method enables virologists to employ an automatic CNN-based segmentation tool for detecting SARS-CoV-2 and demonstrates superiority over GVF and PIG.

## 1. Introduction

Coronavirus disease 2019 (COVID-19), caused by the SARS-CoV-2, was first identified in late 2019 and rapidly evolved into a global public health emergency [1,2,3]. As of April 2026, more than 7.1 million confirmed deaths have been reported worldwide according to the World Health Organization, although the broader societal impact extends beyond mortality alone. Specifically, the COVID-19 pandemic has placed unprecedented pressure on healthcare systems, disrupted global economies, and necessitated the rapid development of diagnostic and analytical tools for viral detection and monitoring [2,3]. In this context, efficient and accurate automatic classification and segmentation of SARS-CoV-2 in medical imaging data are essential for supporting diagnostic workflows and optimising clinical resource allocation.

**Figure 1:**
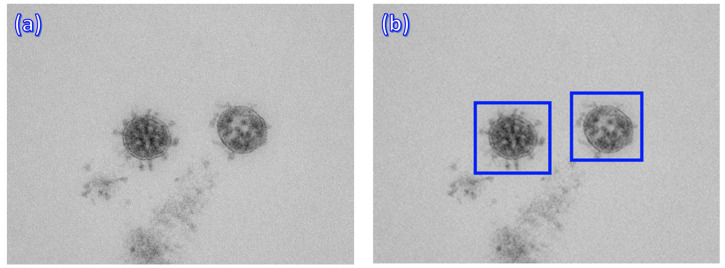
(**a**) Microscopic images of SARS-CoV-2. (**b**) Partial SARS-CoV-2 detection.

Structurally, SARS-CoV-2 exhibits morphological variability in microscopy images and is characterised by four principal structural proteins, spike (S), envelope (E), membrane (M), and nucleocapsid (N), which play critical roles in viral entry, assembly, and replication [4,5,6] (Figure 1). The rapid release of the viral genomic sequence in early 2020 enabled the timely development of reverse transcription polymerase chain reaction (RT-PCR) assays, which remain the gold standard for COVID-19 diagnosis due to their high sensitivity and specificity [7,8,9]. These assays facilitate the detection of infected individuals and also estimate viral load, which is crucial for clinical decision-making and epidemiological surveillance.

Electron microscopy is a critical imaging modality for SARS-CoV-2 research because it offers direct observations of viral morphology and spatial distribution within host cells, thereby generating high-resolution datasets that are essential for developing and validating automated image segmentation methods [10,11]. However, manual analysis of EM images is time-consuming, subjective, and dependent on expert interpretation. Consequently, there is a growing need for automated and reliable image analysis methods capable of accurately detecting and segmenting viral particles.

In recent years, deep learning and image processing techniques have significantly advanced virus detection and classification tasks. In terms of cell segmentation, Constantin et al. employed a seeded watershed algorithm (StartDist) for the nucleus with boundary predictions from a U-Net [12]. Early studies explored deep learning models such as R-CNN, Faster R-CNN, YOLO, and SSD for virus detection in EM images [13]. Similarly, transfer learning approaches employing architectures such as ResNet, DenseNet, and Inception have demonstrated promising performance in virus classification tasks [14,15]. The availability of benchmark datasets and improved computational models has further accelerated research in this domain [16].

More recent studies have focused on segmentation and fine-grained analysis of SARS-CoV-2. For instance, Rodríguez et al. [17] proposed contrast enhancement and segmentation techniques for high-resolution microscopy images, while Goswami et al. [18] combined phase imaging with deep learning for label-free virus detection. Superpixel-based methods have also gained attention—particularly for capturing local structural information in microscopy images.

Potter et al. showed a portable imaging system, described as a biosensor, designed to detect SARS-CoV-2. This system employs computational imaging combined with deep learning for quantification via CNNs [19]. Taha et al. [20] focused on characterising the morphological features of SARS-CoV-2 in TEM images—specifically examining envelope diameter, spike length, roundness, circularity, and area size for identification. In a subsequent study, Taha et al. [21] proposed a novel superpixel algorithm to estimate and determine the density of SARS-CoV-2 spike proteins. This approach aimed to provide insights into the virus’s life cycle, mutations, and disease progression.

Beyond imaging-based approaches, complementary diagnostic techniques such as optical biosensors [22], Raman spectroscopy [23], and point-of-care testing systems [24] have also contributed to SARS-CoV-2 detection. Furthermore, recent research has investigated morphological differences between SARS-CoV-2 and related viruses using EM imaging [25] as well as cross-scale microscopy analysis enabled by machine learning [26]. These developments underscore the interdisciplinary nature of modern virus detection and analysis.

Taha et al. [27] introduced superpixel segmentation techniques for estimating spike protein density and improving virus detection accuracy. Sikder et al. [28] obtained a peak testing accuracy of 97.44% and a quadratic weighted kappa of 0.9719 for heterogeneous virus classification from TEM images. Other work [29] describes methods used in diagnostic electron microscopy in addition to different applications including COVID-19.

Advances in artificial intelligence have also led to the development of more sophisticated segmentation approaches. Few-shot learning methods have been proposed to address data scarcity in EM image segmentation [30], while hybrid deep learning models combining convolutional and sequential architectures (e.g., CNN-BiLSTM) have demonstrated improved performance in related biomedical applications [31]. Additionally, recent work has explored the integration of large-scale models and multimodal approaches such as ViruSeg, which leverage large language-image models to enhance virus image segmentation [32]. Comparative studies using advanced architectures such as ResNet152V2 further highlight the growing importance of deep learning in electron microscopic virus analysis [33]. For example, Taha et al. used transmission electron microscopy (TEM) images of SARS-CoV-2 to estimate the density of the virus’ spike proteins by applying segmentation based on the superpixel technique [34].

However, despite recent progress in microscopy-based SARS-CoV-2 image analysis, an important scientific problem remains unresolved: how to achieve accurate and robust automatic semantic segmentation of SARS-CoV-2 particles in electron microscopy images—particularly when virus boundaries are ambiguous, image contrast is heterogeneous, and particle morphology varies. Addressing this problem is essential for reducing manual workload and improving the reliability of virus identification in practical microscopy systems.

Accordingly, the goal of this study is to develop and validate a cascade semantic segmentation method based on convolutional neural networks combined with super-Euclidean pixel processing for the automatic delineation of SARS-CoV-2 in microscopy images. To achieve this goal, the study addresses the following tasks: (1) prepare and annotate a microscopy image dataset of SARS-CoV-2 particles for segmentation experiments; (2) design a two-stage CNN architecture with an intermediate super-Euclidean pixel processing step; (3) apply the proposed method to automatically segment SARS-CoV-2 particles; (4) compare its segmentation performance with two conventional methods, gradient vector flow (GVF) [35] and Poisson inverse gradient (PIG) [36]; and (5) evaluate the methods quantitatively using expert annotations as ground truth and standard segmentation metrics.

In this way, the study aims to determine whether the proposed cascade CNN framework provides a more accurate and effective solution for SARS-CoV-2 segmentation in microscopy images. This paper is organised as follows: Section 2 describes the applied methods—CNN, GVF, and PIG. Section 3 analyses the experimental results, while Section 4 presents the conclusion.

## 2. Materials and Methods

In this section, we describe the procedure and methodology used for segmenting SARS-CoV-2 in microscopic images (Figure 1a) through semantic segmentation. The first phase of this study involved analysing a set of microscopic images to detect SARS-CoV-2 using the circular object detection technique [37]. This approach approximates the SARS-CoV-2, resulting in a rectangular window (Figure 1b) that indicates where the COVID-19 has been detected. The evaluation of these windows relies on their precision, as they classify only objects that fit within them. To enhance clarity in object detection, semantic segmentation is employed.

This study primarily focuses on the automatic identification of SARS-CoV-2 through the proposed method. It employs a cascade semantic segmentation CNN combined with image super-Euclidean pixel processing to enhance the segmentation’s alignment with the medical GT. The primary objective is to develop and compare the semantic segmentation approach using CNN against the GVF and PIG segmentation methods. This comparison aims to verify the accuracy and efficacy of the proposed method in the specific context of images from a practically applied microscopic system [21].

The first and second parts involve segmentation using GVF and PIG methods, detailed in Section 2.1.1 and Section 2.1.2, respectively. Following this, Section 2.2 presents the proposed image processing application and the implementation of CNNs. The subsequent section evaluates the performance of these three techniques. A dataset of 679 microscopic images containing SARS-CoV-2 was utilised for this purpose (Figure 1a), with a total of 1402 viruses identified (Figure 1b). Initially, object detection was performed using the circular Hough transform [38], and the results were further refined using CNN, GVF, and PIG methods.

**Figure 2:**
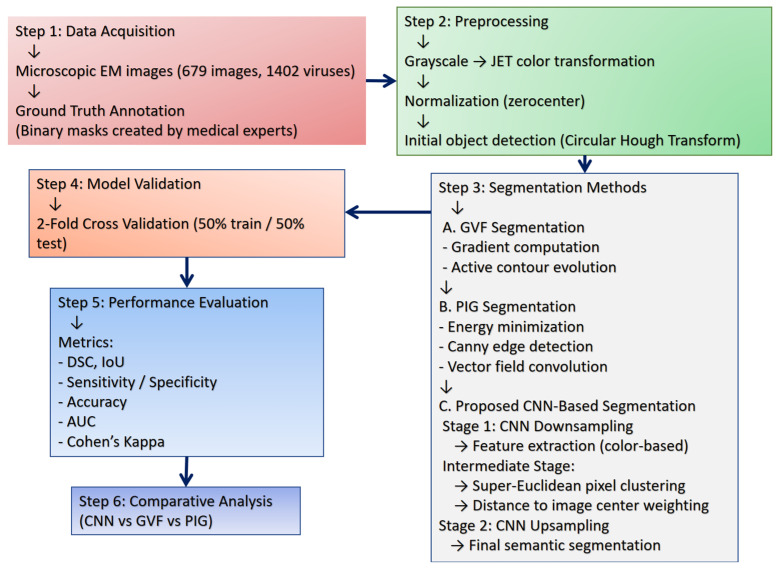
Methodological workflow and research algorithm.

Figure 2 illustrates the methodological workflow of the proposed study. The analytical base consists of 679 microscopic images containing 1402 SARS-CoV-2 instances annotated by medical experts to generate ground truth masks. The methodology includes preprocessing (colour transformation and normalisation), initial object detection using the circular Hough transform, and segmentation using three approaches: GVF, PIG, and the proposed cascade CNN with super-Euclidean pixel processing. The dataset was then evaluated using 2-fold cross-validation, and the results were assessed using standard segmentation metrics derived from the confusion matrix. This structured pipeline ensures reproducibility and allows a direct comparison between classical and deep learning-based methods. The pipeline includes data acquisition and expert annotation; preprocessing and object detection; and segmentation using GVF, PIG, and the proposed CNN-based method with super-Euclidean pixels followed by 2-fold cross-validation and performance evaluation using standard metrics.

### 2.1. Segmentation Methods

This section explains the GVF and PIG segmentation techniques, as well as the proposed semantic segmentation method that utilises CNN.

#### 2.1.1. Gradient Vector Flow

**Figure 3:**
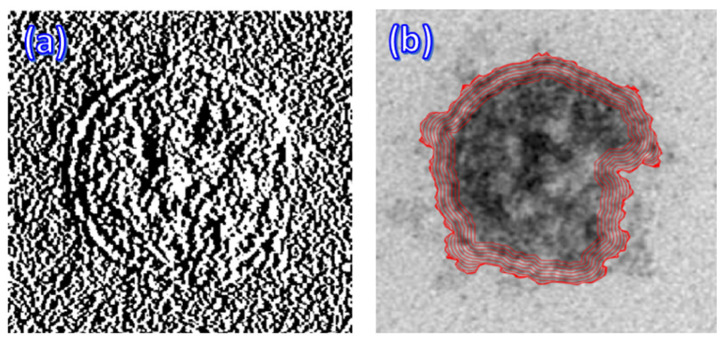
GVF segmentation. (**a**) Gradient. (**b**) Result segmentation.

GVF is applied to images of COVID-19 in a grayscale model (Figure 3). GVF offers more precise initialisation and detection of contours, which often include concavities and convexities—characteristics typical of microscopic images of the SARS-CoV-2.

To calculate the GVF, an edge map function or gradient is first calculated using a Gaussian function (Figure 3a); GVF is applied to obtain [u,v]; u corresponds to dF/dx, the differences in x (horizontal) direction; and v corresponds to dF/dy, the differences in y (vertical) direction (F is the input microscopic image of the SARS-CoV-2).

The curves v(s)=[x(s),y(s)],s∈[0,1] are defined within the image domain as contours; they can be modified and defined under the influence of internal forces from the curve itself and external forces from the image data. Refs. [35,39] proposed an improved contour to obtain a better segmented image (Figure 3b).

The initial parameter values are informed by prior knowledge and several experiments related to SARS-CoV-2. The elasticity parameter, α, controls the boundary strain when combined with the first derivative term (α = 0.50). The stiffness parameter, β, manages the boundary when combined with the second derivative term (β = 0.001). The viscosity parameter, γ, specifies the step size (γ = 1.00). κ functions as a scaling factor for the external force weight term (κ = 0.5). The weighting factor, wEline, is applied to the intensity-based potential term (wEline = 0.01). The weighting factor, wEedge, pertains to the edge-based potential term (wEedge = 0.40). Additionally, the weighting factor, wEterm, is associated with the termination potential term (wEterm = 0.01). The contour position will be calculated over a specified number of iterations (itt = 50).

With the defined parameters, an edge map function and an approximation of its gradient are first applied (fx,fy define the external force field GVF_u and GVF_v), calculating GVF to guide the contour deformation at the boundary edges. GVF is computed as a spatial diffusion of the gradient, in an image-derived edge map, causing diffuse forces that force the contour away from the object and force vectors attracting it closer to the edges. Its definition is given as the vector field F(x,y)=[u(x,y),v(x,y)] which minimises the energy functional $E=\iint \mu \left(u_{x}^{2}+u_{y}^{2}+v_{x}^{2}+v_{y}^{2}\right)+{\left|\nabla f\right|}^{2}{\left|V-\nabla f\right|}^{2}dxdy$. Using variational calculus, the GVF field can be found by solving the following Euler equation: ${\mu \nabla }^{2}u-\left(u-f_{x}\right)\left(f_{x}^{2}-f_{y}^{2}\right)=0$. These equations are known as the gradient diffusion equations. Figure 3b shows the results of variations in the segmentation of SARS-CoV-2.

#### 2.1.2. Poisson Inverse Gradient

**Figure 4:**
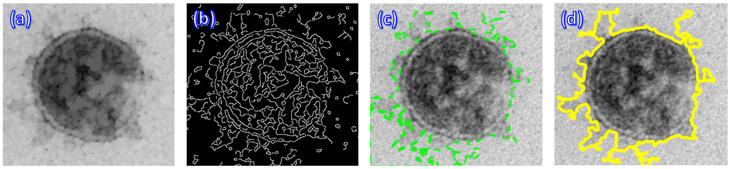
(**a**) Energy minimised of the SARS-CoV-2 image. (**b**) Contour by Canny. (**c**) PIG segmentation first iteration. (**d**) PIG result segmentation.

Based on the active contour model (ACM) [40] and minimising the energy function (E) to identify the contours of SARS-CoV-2 (Figure 4a), the active model is applied via PIG [36,41]. The PIG method estimates the energy field (E) such that the negative gradient of E is the closest vector field to the force field F in the L2 norm sense $E\left(v\right)=\int_{0}^{1}\int_{0}^{1}\left[E_{int}\left(v\right)+E_{ext}\left(v\right)\right]ds$, where v(s) is a surface v(s) = (x(s),y(s)) and E_int and E_ext are the internal and external energies of the image, respectively. The internal energy is defined as $E_{int}=\frac{1}{2}\left(\alpha \sum_{s}{\left|v_{s}\right|}^{2}+\beta \sum_{s}{\left|v_{ss}\right|}^{2}\right)$, where $V_{S}$ and $V_{Ss}$ are the first and second derivatives of the surface v(s), respectively, and α and β are weighting parameters to constrain the degree AC elasticity (1st order) parameter ranges and the AC rigidity (2nd order) of the surface, respectively. For our experiments, we use α = 0.5 and β = 0.1; τ = 0.5 is time step of each iteration.

The edge strength is the gradient of the edge map B of the image; Eext was calculated using the optimised edge map B using a Canny edge detector [42] from the smoothed image (Figure 4b). The external energy represents the edge information of the image, which is defined as $E_{ext}\left(v\right)=-{\left|\nabla \left(G_{\sigma }\left(x,y,z\right)*I\left(x,y,z\right)\right)\right|}^{2}$, where Gσ and I are the Gaussian function and the image, respectively, and ∇ is the gradient operator.

The application of the Vector Field Convolution (VFC) algorithm successfully provided a smooth contour for the internal cross-section within 180 iterations. The initial and final iterations are depicted in Figure 4c and Figure 4d, respectively. Ultimately, the internal cross-sectional area was calculated by integrating the detected contour over the segmented area of the SARS-CoV-2 (Figure 4d).

### 2.2. Semantic Segmentation

Accurate image segmentation is a persistent challenge in computer vision, particularly in biomedical imaging, where low contrast, structural variability, and noise complicate the precise delineation of regions of interest. Consequently, modern approaches increasingly rely on deep neural networks for robust semantic segmentation [43]. In this section, we first describe the preparation of the database utilised for our study. We then detail the architecture of the proposed CNN used for semantic segmentation, which has been enhanced through deep learning techniques [44]. This semantic segmentation is employed to identify SARS-CoV-2 using our two-level CNN deep learning algorithm, incorporating a super-Euclidean pixels method at the intermediate stage. Lastly, we provide a detailed explanation of the development process undertaken to achieve our results, which will be discussed more comprehensively in the following section.

#### 2.2.1. Data Preparation

**Figure 5:**
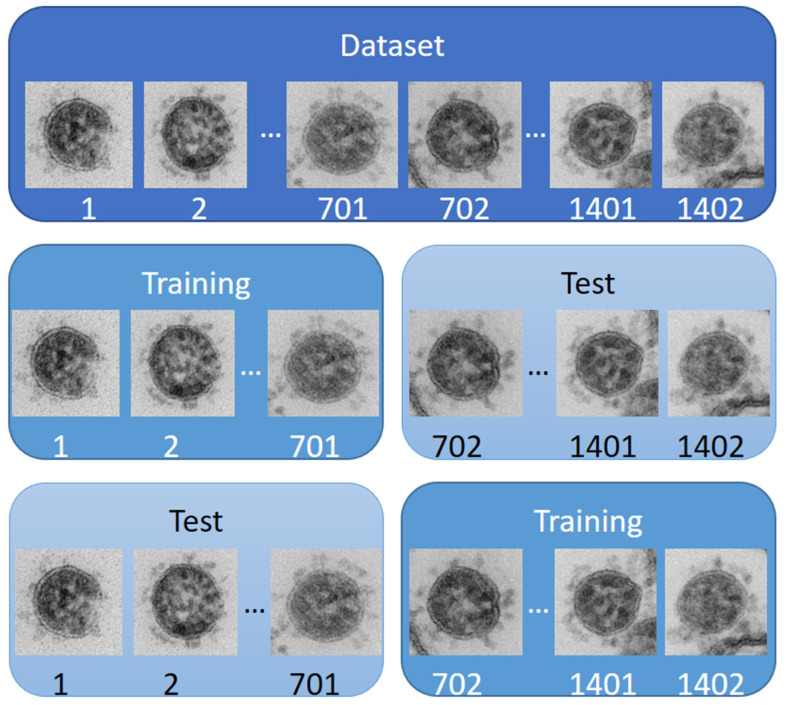
SARS-CoV-2 dataset.

Pixel labelling is conducted by a medical specialist using masks, also known as GT or binary matrices. In this context, the mask serves to identify specific regions in microscopic images where SARS-CoV-2 are located. Pixels representing these regions are assigned a value of 1 (white), whereas pixels with a value of 0 denote the background, which is not relevant to the study. For mask creation, 1402 images of SARS-CoV-2 are available (Figure 5).

To effectively apply CNNs, the input to the first layer must consist of a dataset that will be foundational for training the algorithm. This enables the CNN to learn and subsequently demonstrate its effectiveness in identifying SARS-CoV-2. The dataset is organised with a specific directory structure to facilitate the training and testing of the CNN (Figure 5). The dataset will be partitioned using the k-fold cross-validation (k-fcv) technique, which is a statistical method for evaluating deep learning algorithms in semantic segmentation [45]. In this study, a 2-fold cross-validation approach divides the dataset into two distinct sets (Figure 5).

In this work, the training set comprises half of the dataset (50% Virus images). This set is employed during the training phase to adjust the network parameters. The aim is to ensure that the output layer’s responses closely align with the known data. Test set: Utilised to assess model performance and to obtain a generalisation error that approximates the true error rate. At this stage, both parameters and hyperparameters remain unchanged. In this study, the test set constitutes 50% of the dataset, equating in size to the other subset (the remaining 50% of the Viruses).

In this work, we utilised a database [21] comprising 1402 COVID-19 images, derived from 679 microscopic images, to apply background–foreground segmentation. This process aimed to differentiate the objects in the foreground (foreground–virus) from other elements in the background.

The use of large volumes of training data, or Big Data, for modelling and detecting various types of objects has become increasingly prominent. Notably, the application of convolutional networks has significantly improved object detection tasks in several cases [46,47]. The primary objective is to accurately segment the Virus–Background class. In the following sections, we outline the development of a proposed semantic segmentation algorithm that utilises a CNN in conjunction with image super-Euclidean pixel processing.

#### 2.2.2. Cascade Semantic Segmentation Method by a Convolutional Neural Network

**Figure 6:**
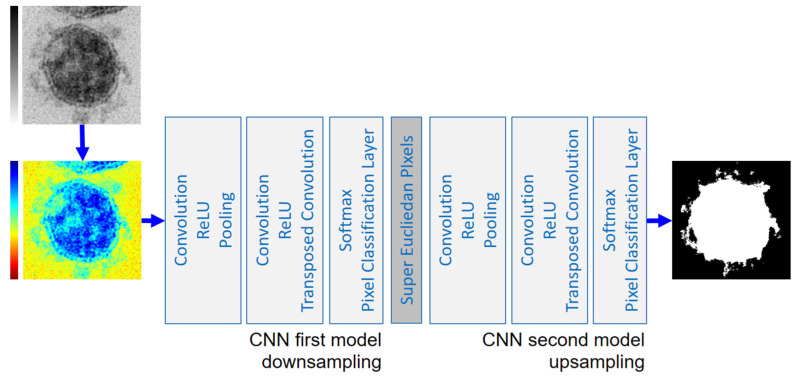
Proposed CNN model (On Supplementary Materials section).

This study aims to develop and deploy a CNN for semantic segmentation utilising a 2-fcv dataset. It employs a straightforward semantic segmentation network comprising a first stage, which involves downsampling through the initial CNN model, and a second stage, which encompasses upsampling via the subsequent CNN model. An intermediate stage utilises the proposed super-Euclidean pixels method. The neural network model applied to this dataset is illustrated in Figure 6. Semantic segmentation [43] is a deep learning algorithm that assigns a specific category and class (e.g., virus or background) to each pixel in an image.

The initial process involves altering the colour space model. Since the image is in grayscale, the goal is to convert it into a three-dimensional colour space. This transformation into a colour image serves as a preliminary step, providing numerical input to the CNN. The grayscale image is transformed using the JET colour model (Figure 6). This conversion into the JET model’s three-dimensional space enables the CNN to filter specific colours, thereby enhancing the segmentation of SARS-CoV-2 compared to inputting the original grayscale image.

**Figure 7:**
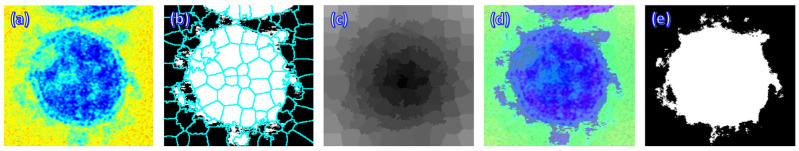
CNN segmentation. (**a**) RGB JET model colour CNN input image. (**b**) Downsampling the first CNN model segmentation. (**c**) Super Euclidean pixel map. (**d**) CNN segmentation. (**e**) Final result segmentation of the upsampling second CNN model.

The RGB (red, green, blue) colour space corresponds to the three orthogonal coordinate axes in 3D space. Grayscale is defined as a linear intensity gradient ranging from black (0,0,0) to white (1,1,1). The colour gradient applied in this study utilises the JET model, which assigns blue (0,0,1) to low values, green (0,1,0) to medium values, and red (1,0,0) to high values. To enhance intuitiveness, cyan (0,1,1) and yellow (1,1,0) are incorporated, ensuring the colour ramp (grey–RGB transformation) navigates solely along the edges of the colour cube, from blue to red (Figure 6). This approach simplifies mapping and speeds up processing while increasing colour variation, thereby facilitating classification and segmentation using CNNs (Figure 7a).

In the first stage, the CNN (downsampling first model) segments the image based on colour characteristics, identifying unwanted parts with similar intensity or colour to the target regions. To enhance the CNN’s accuracy, we introduced an intermediate clustering stage in the system, employing super-Euclidean pixels (Figure 7b). This step retains only the central SARS-CoV-2 area as the region of interest. We then fuse the colour data from the original virus image with the positional information of each cluster relative to the centre. This processed data is subsequently segmented in the second stage of the CNN (upsampling the second model).

The proposed CNNs model initially processes JET colour model images with dimensions of 256 × 256 × 3 (Figure 7a) using ‘zerocenter’ normalisation. It begins with an input convolutional layer comprising 64 convolutions of size 3 × 3, each with a stride of [1,1] and padding of [1,1,1,1]. The ReLU activation function is applied to these convolutions. Subsequently, a pooling layer performs a 2 × 2 max pooling operation, utilising a stride of [2,2] and no padding [0,0,0,0].

A further level of abstraction is achieved by applying a new convolutional layer followed by MaxPooling. This layer utilises 64 3 × 3 convolutions with a stride of [1,1] and padding of [1,1,1,1], combined with the ReLU activation function. Subsequently, a 64 × 4 × 4 Transposed Convolution layer with a stride of [2,2] and output cropping of [1,1] is applied. This is followed by a 2 × 1 × 1 Convolution layer with a stride of [1,1] and padding of [0,0,0,0]. These layers are responsible for transforming the features extracted from the convolutional layers to enable data classification.

The output layer consists of a single neuron employing a softmax activation function. This setup is utilised for binary classification with the Pixel Classification Layer, which applies cross-entropy loss. It is designed to detect regions of the core SARS-CoV-2 of interest, including parts of other SARS-CoV-2 that are not of interest (Figure 7b).

At this stage, we present an accurate and efficient approach for detecting the COVID-19 by analysing the Euclidean distance of superpixels relative to the image centre. Initially, the image in the intermediate layer of the CNN is subsampled. Then, we apply the SuperPixel SILC algorithm [48,49], which uses an input specifying approximately 100 equally sized superpixels, K = 100 (Figure 7b).

The K superpixels are chosen cluster centre $C_{k}\;=\;[R_{k},\;G_{k},\;B_{k},\;x_{k},\;y_{k}]$ with k = [1, K] a regular grid intervals S. For this work, we propose the normalised Euclidean distance measure ($D_{s}$) to be used in the 5D space is defined as $D_{s}=d_{RGB}+(1/S)*d_{XY}$. Where $d_{RGB}=\sqrt{{\left(R_{k}-R_{i}\right)}^{2}+{\left(G_{k}-G_{i}\right)}^{2}+{\left(B_{k}-B_{i}\right)}^{2}}$ from SILC [48,49], and the proposed $d_{XY}=\sqrt{{\left(x_{k}-I_{X}\right)}^{2}+{\left(y_{k}-I_{Y}\right)}^{2}}$ Euclidean distance of each superpixel concerning the centre of the image $\left[I_{X}\;I_{Y}]=[128\;128\right]$ (fixed value).

Following the results of the segmented image (Figure 7b) and the map of the superpixels relative to the image centre (Figure 7c), the final step involves generating the input for the second CNN model used for upsampling. This input is obtained by conducting semantic segmentation on the segmented image (Figure 7b) with superimposed superpixels, as established by the superposition colour map (Figure 7c). To enhance the segmentation results (Figure 7e), retraining of the network is necessary. This should be done using these additional images (Figure 7d) and employing the semantic segmentation network through transfer learning [50,51].

## 3. Evaluation Results

To provide a comprehensive quantitative and qualitative evaluation, the performance of the proposed cascade CNN-based semantic segmentation model was assessed against the GVF and PIG methods using both statistical metrics and visual inspection.

### 3.1. Quantitative Evaluation

**Figure 8:**
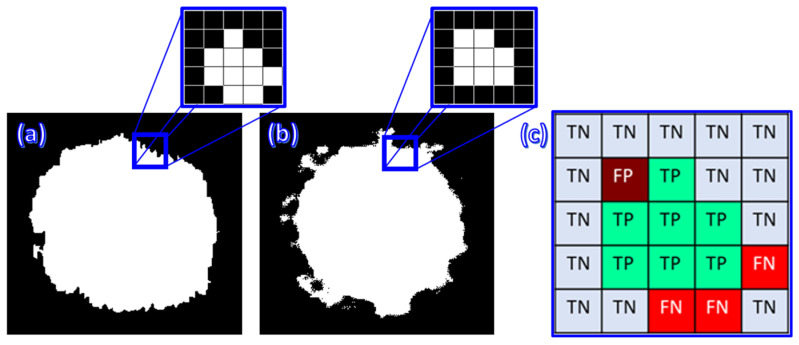
(**a**) Ground truth. (**b**) Predicted binary segmented results. (**c**) Confusion matrix of the overlap between the GT and segmented results.

The performance of semantic segmentation was evaluated by measuring the overlap between the segmented SARS-CoV-2 (using CNN, GVF, and PIG) and the GT. This overlap can also be calculated from the confusion matrix (Figure 8). The evaluation included metrics such as the DSC, intersection over union (IoU), sensitivity, specificity, accuracy, area under the ROC curve, and Cohen’s Kappa. These metrics are based on the number of predicted true positives (TPs) (correctly identified SARS-CoV-2), false positives (FPs) (background incorrectly identified as SARS-CoV-2), true negatives (TNs) (correctly identified background), and false negatives (FNs) (SARS-CoV-2 incorrectly identified as background).

F-measures [52,53] are among the most commonly used metrics for evaluating segmentation performance. These metrics are derived from the sensitivity and precision of a prediction, assessing the overlap between the predicted segmentation and the GT. Precision, in particular, penalises FNs. In this study, we propose using the IoU (or Jaccard index) and the DSC (F1 score or Sorensen-Dice index) as evaluation metrics. The IoU penalises both under-segmentation and over-segmentation more heavily than the DSC does.

Specificity and sensitivity are commonly utilised in medical image processing to assess segmentation and identification performance [52,53]. Sensitivity, also known as recall, measures the ability to accurately detect TPs, such as identifying the region of interest, like the SARS-CoV-2 class. Specificity, on the other hand, evaluates the capability to correctly identify TNs, such as detecting the background.

Accuracy is defined as the ratio of correct predictions—both positive and negative—to the total number of predictions [52,53]. The Receiver Operating Characteristic (ROC) curve represents the relationship between the true positive rate (TPR) and the false positive rate (FPR). Refs. [54,55] are a commonly employed metric for validating the performance of machine learning classifiers. Cohen’s Kappa (Kap) [52,53,54] is a measure of agreement that accounts for chance, used to compare annotated and predicted classifications. Like the AUC score, it adjusts for agreement due to random chance.

**Figure 9:**
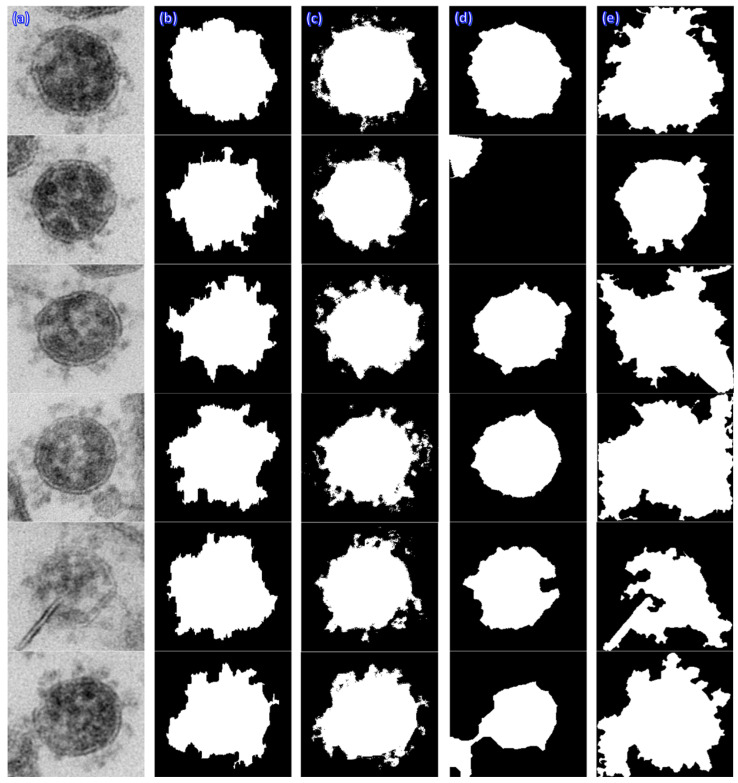
(**a**) SARS-CoV-2. (**b**) Ground truth. (**c**) CNN segmentation. (**d**) GFV segmentation. (**e**) PIG segmentation (On Supplementary Materials section).

The behaviour of these metrics (Table 1 and Figure 9) demonstrates the advantages of semantic segmentation using the proposed method compared to GVF and PIG. These results significantly impact the adoption of the proposed methodology for SARS-CoV-2 detection. The validation with 2-fcv has generated considerable confidence in the proposed work.

The proposed CNN model achieved the following performance (Table 1, mean ± variance) through which the following standard metrics were computed: Dice similarity coefficient (DSC, high overlap with GT); intersection over union (IoU, strong region agreement); sensitivity (recall/TPR, effective virus detection); specificity (TNR, accurate background rejection); accuracy (ACC, high overall correctness); area under the ROC curve (AUC, strong classification capability); Cohen’s Kappa (Kap, excellent agreement beyond chance).

Table 1 presents the outcomes of DSC, IoU, TPR, SPC, ACC, AUC, and Kap for the CNN, GVF, and PIG segmentation results. This analysis reveals that the presented CNN not only achieves higher average metrics but also exhibits lower variance compared to the other two models, GVF and PIG, for segmenting SARS-CoV-2 from microscopic images.

The CNN consistently outperformed GVF and PIG across all metrics. The variance of CNN results is significantly lower, indicating better stability and higher robustness across samples. The largest improvement is observed in DSC and IoU, thus confirming superior segmentation overlap and sensitivity showing improved detection of virus regions.

### 3.2. Qualitative Evaluation

To complement numerical results, qualitative analysis was performed using representative segmentation outputs (Figure 9). Figure 9 displays the results of the semantic segmentation of selected image regions. These segmentation outcomes (Figure 9) corroborate the quantitative findings detailed in Table 1. The segmented images, generated through the proposed CNN method, demonstrate enhanced accuracy in dense regions, including smaller areas. They effectively preserve the details and boundaries of dense tissues.

Table 1 presents the performance metrics for semantic segmentation using the CNN, GVF, and PIG models utilised in this study. Analysis of Table 1 and Figure 9 reveals that the CNN achieved the highest mean accuracy and displayed superior variance in the DSC, IoU, Sensitivity (or recall, also known as the TPR), specificity (or true negative rate), accuracy, and AUC.

Specifically, the CNN achieved the following metrics: a mean DSC of 0.93, a mean sensitivity (recall or TPR) of 0.93, a mean specificity (true negative rate) of 0.95, a mean accuracy (precision) of 0.94, a mean area under the ROC curve (AUC) of 0.94, and a mean Cohen’s Kappa of 0.91. The lowest result was a mean IoU score of 0.87. Although the CNN demonstrated higher global results than other models, our visual segmentation analysis (Figure 9) presents the results separately for each technique to facilitate comparison between models.

The proposed CNN demonstrates more precise boundary delineation of viral structures and improved preservation of fine morphological details, especially in dense viral clusters and small or low-contrast regions. This approach reduced over-segmentation and under-segmentation errors compared to GVF and PIG.

The results are more comprehensively appreciated both visually and qualitatively (Figure 9). These observations are supported by the quantitative data presented in Table 1. Overall, the proposed CNN method demonstrates effective performance. Versus classical methods, GVF struggles with complex concave shapes and produces irregular boundaries. PIG is sensitive to initialization and noise and less consistent in dense regions. CNN (proposed) produces smooth, coherent segmentation masks and better handles heterogeneous textures and intensity variations.

### 3.3. Robustness, Generalisation, and Discussion of Results

The use of two-fold cross-validation (2-fcv) ensures that the model generalises well across different subsets. The reported performance is not biassed toward a specific partition. Furthermore, the integration of super-Euclidean pixel processing improves spatial awareness and central object prioritisation.

The superior performance of the CNN model can be attributed multiple features. First, the cascade architecture (downsampling and upsampling) captures both global and local features. Second, JET colour transformation enhances feature separability. Third, the super-Euclidean pixel intermediate layer introduces spatial weighting relative to the image centre. Finally, deep learning adaptability learns complex virus morphology beyond handcrafted models. The following section expands the discussion.

## 4. Discussion

The results demonstrate that the proposed cascade CNN with super-Euclidean pixel processing provides more accurate and stable semantic segmentation of SARS-CoV-2 particles than the conventional GVF and PIG approaches. Across all evaluated metrics, the CNN achieved the best overall performance including higher DSC, IoU, sensitivity, specificity, accuracy, AUC, and Cohen’s Kappa while also showing lower variance. This indicates not only improved segmentation quality, but also greater robustness across different microscopy samples.

A key reason for this performance gain appears to be the multi-stage design of the proposed framework. First, the grayscale-to-JET transformation enriches the input representation and facilitates feature extraction by the CNN. Second, the intermediate super-Euclidean pixel processing step helps preserve the central virus region and reduces interference from surrounding structures with similar intensity patterns. Third, the cascaded downsampling–upsampling strategy enables the model to capture both local boundary information and broader contextual features, which is especially important in microscopy images where virus contours may be irregular, low-contrast, or partially occluded.

The visual results in Figure 9 support the quantitative findings reported in Table 1. In particular, the CNN generated segmentations with contours that more closely matched the ground truth—especially in dense image regions and in small structural details. In contrast, GVF and PIG were more sensitive to local edge ambiguity and background noise, which may explain their lower overlap metrics and higher variability. These findings suggest that classical contour-based methods remain useful as baselines, but they are less suitable than deep learning approaches for complex SARS-CoV-2 microscopy segmentation tasks.

Despite these promising results, several limitations should be acknowledged. First, the study relied on a dataset derived from 679 microscopy images and 1402 virus instances, which, although sufficient to demonstrate feasibility, remains limited in scale versus contemporary deep learning benchmarks. Second, validation was performed using two-fold cross-validation only; additional folds or external datasets would provide stronger evidence of generalizability. Third, the ground truth masks were produced by expert annotation, which is appropriate, but inter-observer variability was not analysed. Future work should therefore include larger multi-centre datasets, stronger external validation, comparisons with more recent semantic segmentation architectures such as U-Net variants or transformer-based models, and an ablation study to quantify the specific contribution of the JET conversion and super-Euclidean pixel stage.

Overall, the findings confirm that the proposed method is a promising tool for automated SARS-CoV-2 segmentation in microscopy images. Beyond outperforming GVF and PIG, the method shows potential for supporting virologists in faster and more consistent image analysis, which could be useful in future virus morphology studies and related biomedical imaging applications.

### 4.1. Comparative Analysis and Evaluation Results with Recent Studies

A comparison with recent studies indicates that the proposed cascade CNN with super-Euclidean pixel processing performs competitively and, in several respects, favourably relative to prior microscopy-based SARS-CoV-2 image analysis approaches. Importantly, however, the available literature is heterogeneous with respect to imaging modality, analytical target, and evaluation protocol. A substantial portion of recent work has focused on virus classification from transmission electron microscopy (TEM) images rather than pixel-wise semantic segmentation: This limits the possibility of strict one-to-one comparisons.

For example, Matuszewski and Sintorn (2021) introduced a benchmark TEM virus dataset comprising 1245 images from 22 virus classes and reported that a fine-tuned DenseNet201 achieved an F1-score of 0.921 and an accuracy of 93.1% on the test set, thereby establishing a strong classification baseline for TEM virus recognition [16]. Likewise, Ali et al. (2022) reported a customised CNN for 14-class virus classification with 96.1% test accuracy [14], whereas Sikder et al. (2024) obtained a peak testing accuracy of 97.44% and a quadratic weighted kappa of 0.9719 for heterogeneous virus classification from TEM images [28]. These studies demonstrate the effectiveness of deep learning for virus recognition, but they do not address the more demanding task of delineating viral boundaries at the pixel level.

Among studies more closely related to segmentation, Rodríguez et al. (2021) proposed computational enhancement and segmentation procedures for high-resolution SARS-CoV-2 microscopy images, thus emphasising contrast improvement and grayscale regional analysis rather than an end-to-end deep semantic segmentation framework [17]. Goswami et al. (2021) combined label-free phase imaging with deep learning and reported 96% accuracy for distinguishing SARS-CoV-2 from other viruses, but their method addressed particle detection and classification in an optical phase-imaging setting rather than boundary-accurate semantic segmentation in electron microscopy [18]. More recently, a few-shot learning framework for automatic SARS-CoV-2 segmentation in electron microscopy reported a Dice score of 93.3% under a five-shot setting, suggesting that high segmentation quality can be achieved even with limited annotations [30]. In a related TEM study, Tahaa et al. (2024) used superpixel-based segmentation to differentiate SARS-CoV-2 from SARS-CoV and reported a very low RMSE of 0.0275 between automated and human-measured viral areas, thus highlighting the usefulness of region-based morphological analysis [27].

Laue (2025) emphasised that diagnostic electron microscopy remains highly valuable for infectious disease investigation, while also noting practical constraints such as methodological complexity and limited specialist infrastructure [29]. This supports the relevance of automated segmentation tools that can assist expert interpretation in EM-based virology.

Recent reviews also show that virus analysis has expanded far beyond conventional image segmentation alone. John et al. (2023) categorised optical biosensors for virus diagnosis into spectroscopic, nanomaterial-based, and interferometric approaches, while Lee et al. (2023) reviewed major advances in COVID-19 point-of-care testing using lateral flow, vertical flow, microfluidic, and paper-based devices [22,24]. Rumaling et al. (2023) further demonstrated that Raman spectroscopy can provide a label-free biofingerprint for coronavirus detection [23]. These studies are highly relevant to the broader diagnostic landscape, but their main contribution lies in biosensing and rapid detection—not in semantic delineation of viral structures from microscopy images. Accordingly, they offer important context for translational relevance, yet they are not direct competitors to the present segmentation framework.

Another group of studies is more closely related to microscopy-based computational analysis. Petkidis et al. (2023) reviewed machine learning for cross-scale virus microscopy and showed that AI now supports denoising, object segmentation, tracking, classification, and super-resolution across light and electron microscopy [26]. This review is especially useful for positioning the present manuscript because it confirms that segmentation is one component of a wider AI-enabled microscopy pipeline. However, the review paper is a methodological overview rather than an experimentally matched SARS-CoV-2 segmentation benchmark.

More direct comparisons arise from studies cantered on SARS-CoV-2 morphology and TEM analysis. Taha et al. (2023) investigated morphological differences between SARS-CoV-2 and SARS-CoV using TEM images demonstrating the biological importance of extracting quantitative structural descriptors from viral images [25]. In a related study, Taha et al. (2023) estimated SARS-CoV-2 spike-protein density from 586 TEM images using superpixel segmentation [34]. They reported mean spike-density values for SARS-CoV-2 and SARS-CoV, thus showing that region-based segmentation can support quantitative morphometry [34]. These studies are highly relevant because they confirm the utility of superpixel-based intermediate representations in virological image analysis, which conceptually aligns with the super-Euclidean pixel stage proposed here. However, their emphasis is on morphometric estimation and comparative structural analysis rather than full semantic segmentation evaluated with overlap, agreement, and classification metrics such as Dice, IoU, AUC, and Cohen’s kappa.

The literature also includes recent deep learning studies that move closer to the present task. Xiao et al. (2024) proposed automatic SARS-CoV-2 segmentation in electron microscopy based on few-shot learning, thus showing that strong segmentation performance can be obtained even when only limited annotations are available [30]. Wang et al. (2025) introduced ViruSeg—a segmentation framework based on a large language-image model and data augmentation that explicitly targets challenges such as limited labelled virus images, major morphological variability, and indistinct boundaries [32]. These works are particularly important because they represent more contemporary segmentation-oriented baselines. In this context, the present study remains competitive: Its Dice similarity coefficient of 0.9345 is very close to the Dice performance reported by Xiao et al., while also providing a broader set of evaluation indicators including IoU, sensitivity, specificity, accuracy, AUC, and Cohen’s kappa, which together give a more complete assessment of segmentation quality.

Other recent works illustrate the broader trend toward hybrid and comparative AI systems for virus-related detection. Alathari et al. (2024) proposed a CNN-BiLSTM model for COVID-19 IgG antibody detection from a fibre-optic dataset, thus indicating that deep learning is being integrated with non-image biosignal platforms as well [31]. Mithun Ram and Vijayan (2025 proceedings) reported an AI-driven virus segmentation and classification study comparing CNN-based processing with ResNet152V2, further reflecting current interest in joint segmentation-classification pipelines [33]. Nevertheless, these studies target different data modalities or broader algorithmic comparisons and are therefore only partially comparable to the present microscopy-based semantic segmentation framework.

To better contextualise the performance of the proposed method, Table 2 compares the present results with representative recent studies on SARS-CoV-2 and TEM-based virus image analysis considering the analytical task, principal methodology, reported performance, and the main advantages and limitations of each approach.

Taken together, the recent literature suggests four main conclusions. First, electron microscopy remains scientifically important for infectious disease investigation, but it benefits from computational support. Second, many recent virus studies prioritise detection, sensing, or classification, rather than pixel-wise segmentation. Third, superpixel- and morphology-based studies support the biological relevance of structured region analysis, which strengthens the rationale for the proposed super-Euclidean intermediate stage. Fourth, newer segmentation models such as few-shot learning and foundation-model-inspired approaches provide valuable contemporary baselines, yet the present study still demonstrates strong performance through a balanced combination of Dice (0.9345), IoU (0.8782), sensitivity (0.9373), specificity (0.9517), accuracy (0.9449), AUC (0.9446), and Cohen’s kappa (0.9137). Thus, the main contribution of the present manuscript is not merely that it achieves high numerical performance, but that it does so in a framework specifically designed for semantic delineation of SARS-CoV-2 particles in microscopy images, while remaining interpretable in terms of image preprocessing, region-of-interest selection, and comparison against classical segmentation methods (GVF and PIG).

### 4.2. Future Perspectives

A gap exists between technical accuracy and practical integration into medical workflows. This work demonstrates the methodological potential for user interaction, decision support, time savings for experts, and integration into existing microscopy platforms.

From a clinical and laboratory perspective, the proposed method can be useful as a decision support tool, rather than as a standalone replacement for expert analysis. Automatic viral particle segmentation could reduce repetitive manual workload, accelerate microscopy-assisted evaluation, and improve the standardisation of image interpretation in specialised research and diagnostic settings.

Notwithstanding its promising performance, the proposed method should be interpreted as an important methodological step rather than a definitive solution. Future work should prioritise larger-scale validation, comparison with contemporary deep-learning architectures, explainability, and integration into clinically relevant microscopy workflows.

## 5. Conclusions

The COVID-19 pandemic has been the deadliest in recent years, with mortality rates escalating rapidly in developing countries and globally. Thus, it is crucial to continue analysing and studying the SARS-CoV-2. Leveraging advancements in artificial intelligence will enable the development of varied medical tools, which could ultimately reduce mortality rates in the future.

This study demonstrates the effectiveness of semantic segmentation using the proposed CNN technique with a cascaded architecture to achieve high-accuracy segmentation of the SARS-CoV-2 from microscopic images. The proposed approach incorporates a CNN network comprising a first-stage design, which involves downsampling the initial CNN model, and a second-stage design, which involves upsampling the second CNN model. This architecture offers significant advantages for semantic segmentation.

During the first stage, the model captures features based on the colour of the preprocessed microscopy image of the SARS-CoV-2, employing a transformation from grey-scale to jetRGB. Through the proposed intermediate layer, the super-Euclidean pixel map, the model assigns weight to the position relative to the image centre. In the second stage, the model captures multi-scale features and emphasises important regions of the image.

These cascading enhancements, along with the intermediate layer, result in more accurate segmentation of the SARS-CoV-2. This underscores the advantage and potential of deep learning models compared to classical methods such as GVF and PIG.

Despite these promising results, this study has several limitations. First, the dataset was limited to 679 microscopic images containing 1402 SARS-CoV-2 instances, which may restrict the generalisability of the model to images obtained from other microscopy systems, laboratories, sample preparation protocols, or viral variants. Second, the proposed CNN was validated using two-fold cross-validation; therefore, broader validation using larger, multicentre datasets is required. Third, although the model outperformed GVF and PIG, its performance still depends on the quality of the initial image acquisition, ground-truth annotations, and preprocessing steps including JET colour transformation and super-Euclidean pixel generation.

Future research should focus on expanding the dataset including images from different microscopy platforms and viral strains as well as validation under diverse experimental conditions. Future work should also compare the proposed cascade CNN with more recent deep learning architectures such as U-Net variants, attention-based models, and transformer-based segmentation networks. In addition, explainable artificial intelligence methods should be incorporated to improve clinical interpretability and increase confidence among virologists and medical specialists. Finally, integration of the proposed segmentation system into semi-automatic microscopy workflows could support faster viral morphometry, quantitative image analysis, and future diagnostic research.

## Figures and Tables

**Table 1:** Segmentation metrics.

	Convolutional Neural Network CNN	Gradient Vector Flow GVF	Poisson Inverse Gradient PIG
Dice Similarity Coefficient $DSC=\frac{2TP}{2TP+FP+FN}$	0.9345 ± 0.0006	0.8343 ± 0.0402	0.6931 ± 0.1110
Intersection over Union $IoU=\frac{TP}{TP+FP+FN}$	0.8782 ± 0.0018	0.7504 ± 0.0424	0.6067 ± 0.0911
Sensitivity (Recall) $TPR=\frac{TP}{TP+FN}$	0.9373 ± 0.0018	0.8016 ± 0.0449	0.7756 ± 0.1385
Specificity $SPC=\frac{TN}{TN+FP}$	0.9517 ± 0.0012	0.9458 ± 0.0039	0.8134 ± 0.0201
Accuracy $ACC=\frac{TP+TN}{TP+FP+TN+FN}$	0.9449 ± 0.0004	0.8856 ± 0.0092	0.7969 ± 0.0192
Area Under the ROC Curve $AUC=\frac{1}{2}\left(\frac{TP}{FP+FN}+\frac{TN}{TN+FP}\right)$	0.9446 ± 0.0431	0.8737 ± 0.0124	0.7945 ± 0.0271
Cohen’s Kappa $Kap=\frac{\left(TP+TN\right)-fc}{\left(TP+FP+TN+FN\right)-fc}$ $fc=\frac{\left(TN+FN\right)\left(TN+FP\right)+\left(FP+TP\right)\left(FN+FP\right)}{TP+FP+TN+FN}$	0.9137 ± 0.0011	0.7888 ± 0.0498	0.6136 ± 0.1221

**Table 2:** Comparative analysis of recent studies related to SARS-CoV-2 and virus image analysis.

Reference	Task	Main Method	Main Reported Results	Advantages	Limitations Relative to the Present Study
Ali et al. (2022) [14]	TEM; 14-class virus classification	Custom CNN vs. XceptionNet, MobileNet, DenseNet201	Accuracy = 96.1%	High classification accuracy with comparatively efficient architecture	Multi-class classification; not foreground-background segmentation
Matuszewski & Sintorn (2021) [16]	TEM; multi-class virus classification	Fine-tuned DenseNet201	F1-score = 0.921; Accuracy = 93.1%	Strong TEM benchmark; publicly available dataset	No pixel-level segmentation
Goswami et al. (2021) [18]	Phase imaging; virus detection/classification	Deep learning + computational specificity	Accuracy = 96%; Inference time ≈ 60 ms/image	Fast, label-free detection	Different modality (optical, not EM); not segmentation
John et al. (2023) [22]	Virus diagnosis/biosensing	Optical biosensor review	No unified metric (review study)	Broad diagnostic coverage	No segmentation results
Rumaling et al. (2023) [23]	Coronavirus detection	Raman spectroscopy biofingerprint	High sensitivity detection (qualitative; no standardised accuracy reported)	Label-free, rapid detection	Not microscopy-based segmentation
Lee et al. (2023) [24]	COVID-19 POCT diagnostics	Review of microdevice-based testing	No unified quantitative metric (review)	High clinical relevance	Not image segmentation
Taha et al. (2023) [25]	TEM virus morphology	Morphological feature extraction	Quantitative descriptors (diameter, spike length, circularity)—no Dice/IoU	Biologically meaningful analysis	No segmentation evaluation metrics
Petkidis et al. (2023) [26]	Cross-scale microscopy	AI review (segmentation, tracking, etc.)	No specific benchmark values (review)	Strong conceptual AI framework	Not a direct experimental comparison
Tahaa et al. (2024) [27]	TEM segmentation/morphometry	Superpixel segmentation	RMSE = 0.0275 (area estimation error)	Accurate morphometric agreement	No overlap metrics (Dice/IoU/AUC)
Sikder et al. (2024) [28]	TEM virus classification	Functional deep learning model	Accuracy = 97.44%; QWK = 0.9719	Very high classification performance	Not segmentation
Laue (2025) [29]	Diagnostic EM	Review of EM applications	No numerical ML metrics (review)	Strong methodological relevance	No automated segmentation results
Xiao et al. (2024) [30]	EM SARS-CoV-2 segmentation	Few-shot learning	Dice ≈ 0.933 (93.3%)	Direct segmentation comparison	Few-shot setup; limited metrics reported
Alathari et al. (2024) [31]	Antibody detection	CNN-BiLSTM (fibre-optic data)	High classification performance (exact accuracy varies by setup; ~95–98%)	Multimodal deep learning	Different modality (non-image segmentation)
Wang et al. (2025) [32]	Virus segmentation	Large language-image model (ViruSeg)	Improved segmentation performance (no standardised public Dice reported yet)	Modern foundation-model approach	Not directly comparable dataset
Mithun Ram & Vijayan (2025) [33]	Segmentation + classification	CNN vs. ResNet152V2	Improved accuracy vs. baseline CNN (quantitative gains reported comparatively)	Hybrid evaluation	Limited SARS-CoV-2-specific benchmarking
Taha et al. (2023) [34]	Spike density estimation	Superpixel segmentation	Quantitative spike density estimation (no Dice/IoU)	Strong biological interpretability	Not full semantic segmentation
Present study (2026)	SARS-CoV-2 semantic segmentation	Cascade CNN + JET + super-Euclidean pixels	DSC = 0.9345; IoU = 0.8782; TPR = 0.9373; SPC = 0.9517; ACC = 0.9449; AUC = 0.9446; Kap = 0.9137	Pixel-level segmentation; comprehensive metrics; superior to GVF and PIG	Requires external validation and broader benchmarking

## Data Availability

The original contributions presented in this study are included in the article/Supplementary Material. Further inquiries can be directed to the corresponding author.

## Supplementary Materials

The following supporting information can be downloaded at https://drive.google.com/drive/folders/1Tp3PNugibYZGxZnuNPuH_UlfFKsrLZKW?usp=sharing (accessed on 13 August 2025). The information is distributed in 7 folders. 1Figures: original images of Figure 1, Figure 2, Figure 3, Figure 4, Figure 5, Figure 6 and Figure 7 in ppt format; 2MicroscopicImageVirus: Original SARS-CoV-2 microscopy images; 3GroundTruthImages: Ground truth of SARS-CoV-2 microscopy images for artificial intelligence applications; 4CodeVirusDL: MATLAB Deep Learning segmentation code; 5CodeVirusGVF: MATLAB (MathWorks, Natick, MA, USA) Gradient Vector Flow segmentation code; 6CodeVirusPIG: MATLAB Poisson Inverse Gradient segmentation code; 7ResultsVirusSegmentation: Segmentation results of the SARS-CoV-2 microscopy images extracted from the CNN, GVF, PIG.

## Abbreviations

The following abbreviations are used in this manuscript:

ACC	accuracy or precision
ACM	active contour model
AUC	area under the ROC curve
CNNs	convolutional neural networks
COVID-19	coronavirus disease 2019
DSC	dice similarity coefficient
E	envelope
E	energy function
EM	electron microscopy
FNs	false negatives
FPR	false positive rate
FPs	false positives
GT	ground Truth
GVF	gradient vector flow
IgG, IgA, and IgM	antibodies
IoU	intersection over union
itt	iterations
Kap	Cohen’s Kappa
k-fcv	k-fold cross-validation
M	membrane
N	nucleocapsid
PIG	poisson inverse gradient
ROC	receiver operating characteristic
RT-PCR	reverse transcription polymerase chain reaction
S	spike
SARS-CoV-2	severe acute respiratory syndrome coronavirus 2
SILC	simple linear iterative clustering
SoA	state of the art
SPC	specificity or true negative rate
TNs	true negatives
TPR	sensitivity, Recall, or true positive rate
TPs	true positives
VFC	vector Field Convolution
wEedge	edge-based potential term
wEline	intensity-based potential term
A	elasticity parameter
B	stiffness parameter
Γ	viscosity parameter
K	κ functions
